# Sex Differences in Human Ankle Stiffness During Standing Balance

**DOI:** 10.3389/fspor.2020.570449

**Published:** 2020-10-09

**Authors:** Ermyntrude Adjei, Varun Nalam, Hyunglae Lee

**Affiliations:** ^1^School of Biological and Health Systems Engineering, Arizona State University, Tempe, AZ, United States; ^2^School for Engineering of Matter, Transport and Energy, Arizona State University, Tempe, AZ, United States

**Keywords:** ankle stiffness, sex differences, gender differences, ankle injury, human ankle, ankle impedance, standing balance

## Abstract

The purpose of this study is to quantify sex differences in 2-dimensional (2D) ankle stiffness during upright standing balance and investigate the mechanisms for the differences. A dual-axis robotic platform, capable of perturbing the ankle and measuring the corresponding ankle torques in both the sagittal and frontal planes, was used to reliably quantify the 2D ankle stiffness while healthy young human subjects perform a range of standing balance tasks, specifically, ankle muscle co-contraction tasks, weight-bearing tasks, and ankle torque generation tasks. In all task conditions and in both planes of ankle motion, ankle stiffness in males was consistently greater than that in females. Among all 26 experimental conditions, all but 2 conditions in the frontal plane showed statistically significant sex differences. Further investigation on the normalized ankle stiffness, scaled by weight times height, suggests that while sex differences in ankle stiffness in the sagittal plane could be explained by sex differences in anthropometric factors as well as neuromuscular factors, the differences in the frontal plane are mostly explained by anthropometric factors. This study also demonstrates that the sex differences in the sagittal plane were significantly higher as compared to those in the frontal plane. The results in this study will provide a foundation for not only characterizing sex differences in ankle stiffness during locomotion, but also investigating sex differences in lower body stability and risk of ankle injury.

## Introduction

The human ankle is an essential joint which plays one of the most important roles in postural stability and locomotion (Winter, 1995). It contributes to the movement and stabilization of the entire human body in both static and dynamic conditions (Robertson and Winter, 1980). Despite the crucial roles of the ankle in lower extremity function, the incidence of musculoskeletal injuries at the ankle joint is an ever-increasing problem. Notably, it has been reported that the incidence of ankle injuries in females is significantly higher than in males engaging in similar activities, such as basketball and soccer (Elias, 2001; Ristolainen et al., 2009).

The higher risk of musculoskeletal injuries in females has been attributed to anatomical, hormonal, and neuromuscular factors that differentiate females from males. Anatomically, the increased rate of musculoskeletal injury is largely associated with the greater range of motion (Beynnon et al., 2001), lower Young's modulus (Kubo et al., 2003), and higher joint and ligamentous laxity (Wilkerson and Mason, 2000) in females. It is also speculated that cyclic hormonal variations could cause a decrease in the strength of muscles and ligaments, and could increase in ligamentous laxity and a decrease in stability (Hewett, 2000). Further, sex differences in neuromuscular control to properly resist external loading and stabilize the body could contribute to sex differences in musculoskeletal injuries (Granata et al., 2002a,b).

Compared to the substantial research on sex differences in knee injuries and their underlying mechanisms (Hewett, 2000; Granata et al., 2002a,b), there is very limited study investigating factors contributing to sex differences in ankle injuries, in particular the neuromuscular factor. In an effort to better understand the higher risk of ankle injury in females, this paper investigates sex differences in ankle stiffness, one of the most important neuromuscular factors that resist external loading and hence prevent ankle injury (Winter, 1995; Brockett and Chapman, 2016).

Given the importance of ankle stiffness in lower extremity function, it has been extensively studied for the past decades, but most studies have focused on characterizing ankle stiffness in a single plane of movement (Kearney et al., 1990; Rouse et al., 2014). More recent studies have characterized 2D ankle stiffness in both the sagittal and frontal planes, since it contributes to not only dorsiflexion-plantarflexion (DP) movement in the sagittal plane but also inversion-eversion (IE) movement in the frontal plane (Roy et al., 2009; Lee et al., 2011, 2014b; Martelli et al., 2019). These studies showed that ankle stiffness is highly direction dependent, being significantly higher in DP in the sagittal plane than in IE in the frontal plane. However, the characterization using wearable ankle robots was strictly limited to non-functional seated tasks. In order to overcome this limitation, robotic platforms, capable of applying perturbations to the ankle, have been used to characterize 2D ankle stiffness during standing balance (Ficanha et al., 2016; Nalam and Lee, 2019). These studies have demonstrated that ankle stiffness in the sagittal plane is significantly higher than in the frontal plane even during standing balance, but the difference is more pronounced than that in the seated studies (Nalam and Lee, 2017, 2018; Ribeiro et al., 2018).

However, there is still little information regarding the sex differences in ankle stiffness. Only one study investigated sex differences in 2D ankle stiffness in the IE-DP space, but it was limited to a static seated position (Trevino and Lee, 2018). While this study has provided an important baseline to understand sex differences in ankle stiffness, it is unknown if results obtained in the non-functional seated tasks would be applicable to functional tasks, such as standing balance and walking. As standing is fundamental in everyday activities and serves as a precursor to the initiation of other activities of daily living, identifying the sex differences in ankle stiffness during upright standing balance is significant.

This paper aimed at investigating sex differences in 2D ankle stiffness during upright standing balance. The differences were quantified under various task conditions, specifically, varying ankle muscle co-contraction, weight-bearing, and ankle torque generation tasks. This study also investigated sex differences of normalized ankle stiffness, scaled by weight times height, to determine how anthropometric factors influence the sex differences in 2D ankle stiffness.

We hypothesized that ankle stiffness in females is significantly lower than in males in both the sagittal and frontal planes and in all task conditions. We also hypothesize that the sex difference in ankle stiffness in the sagittal plane is significantly higher than that in the frontal plane, because the degree of ankle stiffness modulation is substantially higher in the sagittal plane than the frontal plane (Lee et al., 2014a,c; Lee and Hogan, 2015). In addition, based on previous findings of sex differences in active muscle mechanics (Granata et al., 2002a), we further hypothesized that the sex difference in ankle stiffness in the sagittal plane still persists even after normalization by body weight times height, but not in the frontal plane.

## Materials and Methods

### Subjects

Twenty young men (age: 20–27 years; weight: 60.7–88.0 kg; height: 154.5–179.2 cm) and twenty young women (age: 18–32 years; weight: 44.0–82.1 kg; height: 155.5–177.7 cm) with right limb dominance, and with no reported history of musculoskeletal or connective tissue disorders that could affect ankle stiffness were recruited among students at Arizona State University (ASU). Separate independent *t*-tests showed that there was no significant difference in age (*p* = 1.0), but there were significant differences in both height and weight (*p* < 0.001). All protocols were approved by the ASU Institutional Review Board (STUDY00010123). Subjects gave written informed consent before participation.

### Experimental Setup

To quantify 2D ankle stiffness during upright standing balance, specifically in both the sagittal and frontal planes, we used a novel dual-axis robotic platform (Nalam and Lee, 2019). It is capable of applying rapid position perturbations to the ankle in the sagittal and frontal planes and measuring the corresponding ankle torques using a force plate (9260AA3, Kistler, NY) embedded in the platform.

**Figure 1:**
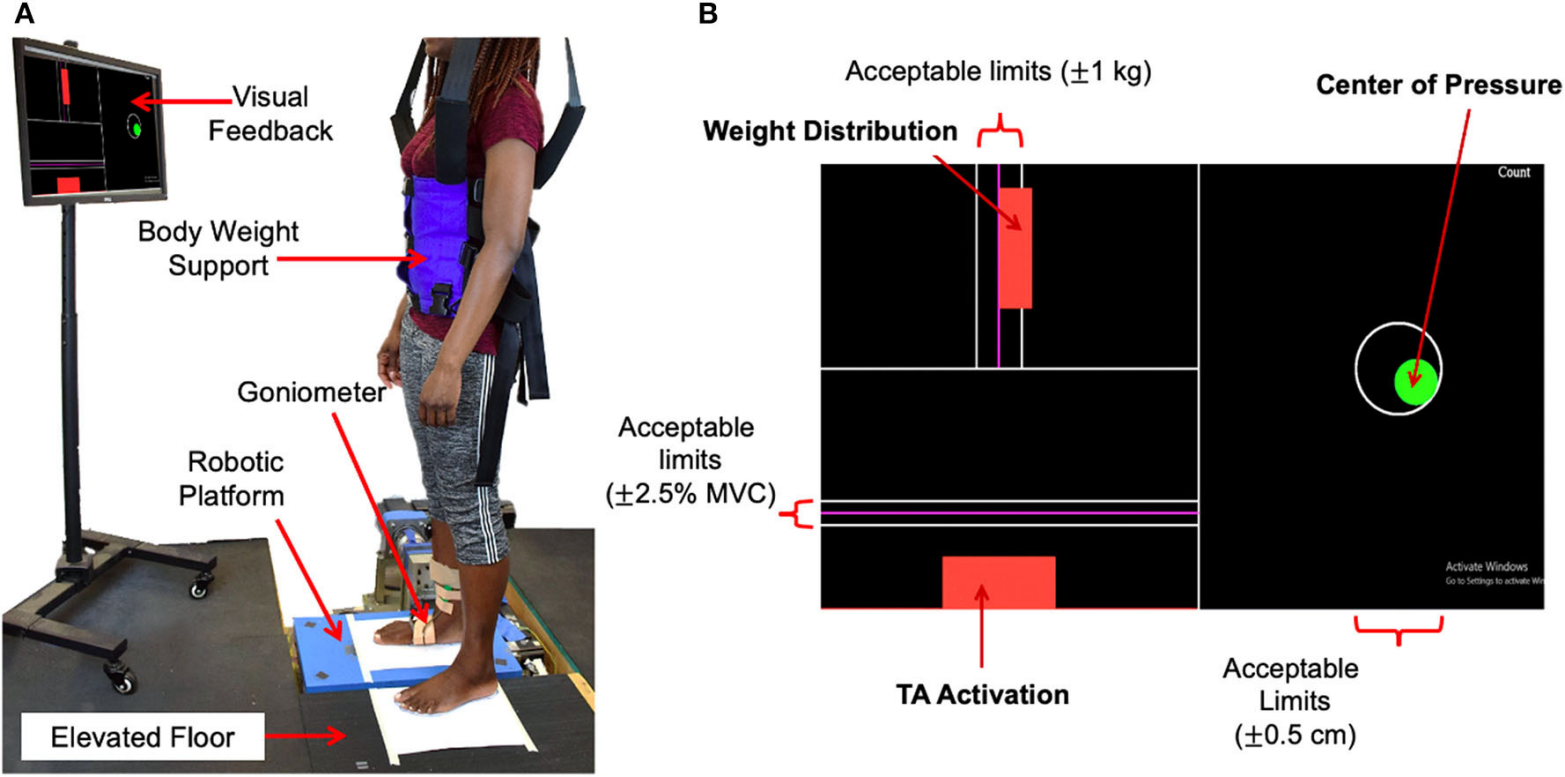
Experimental setup. **(A)** Standing balance setup, **(B)** visual feedback showing the target, current levels, and acceptable limits of 3 parameters to be controlled during standing balance tasks.

Subjects stood upright with their right foot on the robotic platform, recessed into an elevated floor, and their left foot on the elevated floor to the left of the recessed platform (Figure 1A). The right foot was placed in a fashion to ensure that the axis of rotation of the robotic platform for DP was in line with that of the ankle. The axis of rotation of the robotic platform for IE was about 10 cm below that of the ankle, but our previous study confirmed that this offset has minimal impact on IE ankle stiffness estimation (Nalam and Lee, 2019).

In addition to the robotic platform, the experimental setup consisted of a dual-axis goniometer, surface electromyography (EMG) sensors, a safety harness, and a visual feedback display. Ankle angles in both the sagittal and frontal planes, i.e., DP and IE angles, were measured using the electro-goniometer (SG 110, Biometrics, Ltd., UK) attached to the ankle-foot complex. While the goniometer specification for repeatability is 1° for the range of 90°, its measurement error for 3° rotation was very small because the error of the strain gauge based sensor scales linearly with the range of motion. This was validated by comparing encoder measurements in the platform with measurements of the goniometers attached to the platform along the sagittal and frontal planes. The measurement error in both planes of motion for sinusoidal perturbations of varying frequencies (0.5–2 Hz) and amplitude 3 was 0.07 ± 0.008. Muscle activation of major ankle muscles, specifically, tibialis anterior (TA), soleus (SL), medial gastrocnemius (MG), and peroneus longus (PL), was measured using wireless surface EMG sensors (Trigno EMG systems, Delsys, MA). These EMG sensors were positioned according to the SENIAM recommendations (Merletti and Hermens, 2000). To ensure safety, each subject wore a safety harness attached to a bodyweight support system (LiteGait, AZ), but no body weight support was provided. The visual feedback display was placed in front of the subject at the eye level. It showed the target and current levels of 3 parameters to be controlled during standing balance tasks: weight distribution between the legs, center-of-pressure (CoP) in both DP and IE directions, and TA muscle activation (Figure 1B). Controlling CoP alone allowed the subjects to maintain ankle torque at a given target level. Controlling both CoP and TA activation allowed the subjects to effectively control the level of co-contraction of ankle muscles.

A single board computer (PCM 3356, Advantech, CA) was used to control the setup and acquire data using a real-time Simulink model (Mathworks, MA) at 2 kHz.

### Experimental Protocol

Before main experiments, weight, and CoP during quiet standing (namely neutral CoP) were recorded. In addition, maximum voluntary contraction (MVC) of each of the 4 selected ankle muscles was measured according to the standard muscle testing procedures (Hislop, 2013). These measurements were used as references to determine target levels for three tasks in main experiments.

All subjects participated in two sets of experiments, one for the quantification of stiffness in the sagittal plane (*K*_*sagittal*_) and the other for stiffness in the frontal plane (*K*_*frontal*_). Each experiment consisted of 3 distinct tasks, namely, muscle co-contraction tasks, weight-bearing tasks, and ankle torque generation tasks.

For the muscle co-contraction tasks, ankle stiffness was quantified at 4 different levels of ankle muscle co-contraction: 0 (relaxed), 10, 15, and 20% of the maximum voluntary co-contraction (MVCC). Subjects were instructed to maintain 0 (relaxed), 10, 15, and 20% MVC of TA while keeping the neutral CoP, i.e., 0 cm CoP. Controlling the neutral CoP during TA (dorsiflexor and inverter) activation essentially requires proper counter activation of plantarflexors and evertors, and thus this instruction could properly change the level of overall ankle muscle co-contraction. During this task, subjects were instructed to load 50% of body weight in one leg, which was defined as the neutral weight-bearing.

For the weight-bearing tasks, ankle stiffness was quantified at 4 different levels of weight-bearing: 30, 50 (neutral weight-bearing), 70, and 90% of the total body weight in the right leg. Subjects were instructed to maintain the neutral CoP during this task, but no instruction was given regarding muscle activation.

For the ankle torque generation tasks, we commanded different offsets of the CoP to target different levels of ankle torque. Ankle stiffness was quantified at 5 different levels of CoP offset. In the sagittal plane, −2, 0 (neutral CoP), +2, +4, and +6 cm were tested. In the frontal plane, −1.5, −0.75, 0 (neutral CoP), +0.75, and +1.5 cm were tested. Plantarflexion torque and inversion torque are required to achieve positive CoP in the sagittal and frontal planes, respectively. Subjects were instructed to maintain the neutral weight-bearing condition, but no instruction was given regarding muscle activation.

Each of the 26 experimental conditions [(4 muscle co-contraction levels + 4 weight-bearing levels + 5 CoP or ankle torque levels) × 2 planes of ankle motion] was repeated 15 times. For each trial, subjects were instructed to reach and control the selected target levels displayed on the visual feedback display. Feedback indicators for each task changed from red to green when acceptable limits of the target levels were maintained. The acceptable limits were within ±0.5 cm of the target for CoP, ±2.5% MVC of the target for TA activation, and ±1 kg of the target for weight. These limits were selected to ease subjects' difficulty in maintaining the exact target level while minimizing the change in ankle stiffness due to deviations from the target level. During the weight-bearing and ankle torque generation tasks, visual feedback of muscle activation was disabled.

Once acceptable limits of the target levels were maintained at a random period of 0.5–0.7 s, a rapid ramp-and-hold perturbation lasting for 100 ms with an amplitude of 3 was applied to the ankle. Dorsiflexion and eversion perturbations were used to quantify *K*_*sagittal*_ and *K*_*frontal*_, respectively. Plantarflexion and inversion perturbations were not used because of possible loss of contact between the robotic platform and the foot during perturbations.

For each set of experiment, a total of 195 trials were split into 13 blocks (15 trials/block). Each block contained 15 trials of one of the three tasks, and the order of the target levels within the block was fully randomized. To prevent fatigue during the experiment, at least a 3-min rest period between blocks was provided.

### Data Analysis

Data from the experiments (ankle kinematics, ankle torques, and EMG data) were collected using a data acquisition board (DX-32 AT DAQ; Diamond Systems, CA) at a sampling rate of 2 kHz. Ankle kinematics and torque data were filtered using a 2nd order Butterworth low-pass filter having a cut-off frequency of 20 Hz, while the EMG data was demeaned, rectified, and filtered using a 2nd order Butterworth low-pass filter with a cut-off frequency of 5 Hz. Torques due to platform dynamics were identified under no loading condition (no human subject on the platform) and subtracted from each subject's measured dynamics to obtain the ankle torques for each subject.

Ankle stiffness was calculated by fitting a 2nd order model, consisting of ankle stiffness, ankle damping, and foot inertia, to the measured ankle kinematics and torques for a window of 100 ms starting from the onset of the perturbation. To check the reliability of parameter estimation, the percentage variance accounted for (%VAF) between the estimated ankle torque calculated from the estimated stiffness, damping, and inertia and the measured ankle torque was calculated (Lee et al., 2014c; Lee and Hogan, 2015).

### Statistical Analysis

First, we tested if there exist any significant sex differences in 2D ankle stiffness (*K*_*sagittal*_ and *K*_*frontal*_) and normalized 2D ankle stiffness (*K*_*normalized*_*sagittal*_ and *K*_*normalized*_*frontal*_) for the 3 task conditions (muscle co-contraction, weight-bearing, and ankle torque generation tasks). Normalized ankle stiffness was calculated by dividing the ankle stiffness by the total body weight of the subject times height of the subject.

For each stiffness and each normalized stiffness, we performed a separate mixed-design analysis of variance (mixed ANOVA), with task level as the within-subject factor and sex as the between-subject factor. Following the mixed ANOVA, we performed *post-hoc* analyses by running unpaired, independent, two-tailed *t*-tests to identify sex differences at each task level.

Next, we tested if the sex difference in ankle stiffness and normalized ankle stiffness in the sagittal plane is significantly higher as compared to that in the frontal plane. For each task, we performed a separate mixed ANOVA, with plane of ankle motion as the within-subject factor and sex as the between-subject factor and investigated the significance of interaction between these two factors.

In all statistical analyses, we checked normality of data by running Shapiro–Wilk tests, and evaluated equal variance (homogeneity of variance) across data sets by running Levene's tests. If the null hypothesis is rejected in the Levene's tests, equal variance was not assumed in the subsequent statistical analyses. In addition, Mauchly's test of sphericity was used to formally test the assumption of sphericity. If the assumption was violated, the degrees-of-freedom were adjusted using the Greenhouse–Geisser correction before calculating the *p*-value. All statistical tests were made using the SPSS statistical package (IBM, NY) at a significance level of *p* < 0.05. Asterisks (^***^p < 0.001, ^**^p < 0.01, ^*^p < 0.05) and error bars were used in the result figures to denote statistical difference and mean ±1 standard deviation (SD). The SD was presented in parentheses.

## Results

### Quantification of 2D Ankle Stiffness

**Figure 2:**
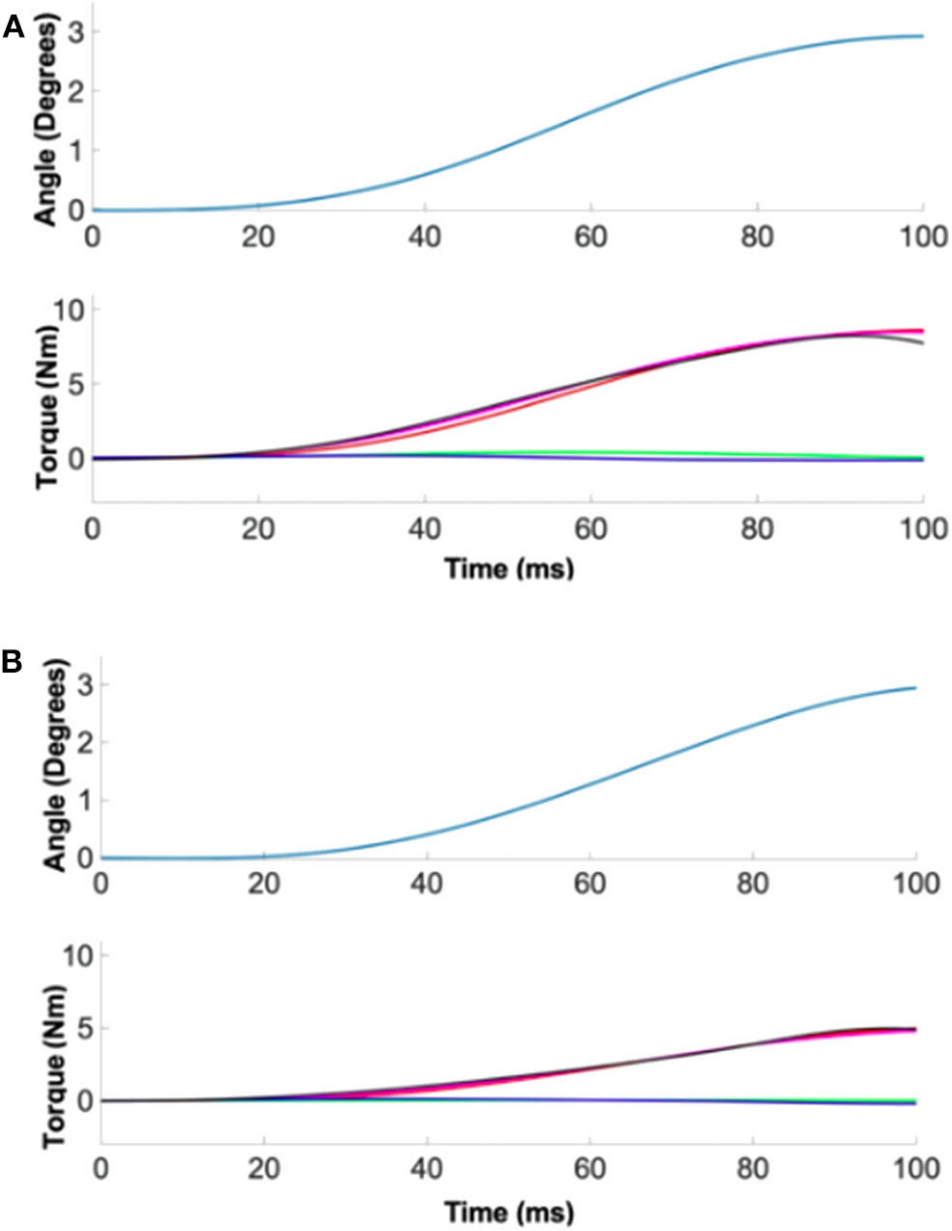
A representative quantification of ankle stiffness by linear regression. (Top) The position perturbation profile. (Bottom) The torque responses. Red, green, and blue denote the torque contribution by ankle stiffness, damping, and inertia. Measured torque (black) matched well with the estimated torque (magenta) by summing the torque contributions of three ankle parameters. **(A)** Male example, **(B)** female example.

Based on precise ankle torque and kinematic measurements, ankle stiffness was quantified with a high reliability in both the sagittal and frontal planes in all 40 subjects (Figure 2). The average %VAF for all 20 male subjects across all 13 experimental conditions was 94.6% (0.3%) and 97.6% (0.6%) in the sagittal and frontal planes, respectively. For all 20 female subjects, the average %VAF was 98.9% (0.3%) and 97.6% (1.5%) in the sagittal and frontal planes, respectively.

### Sex Differences in Ankle Stiffness During Muscle Co-contraction

**Figure 3:**
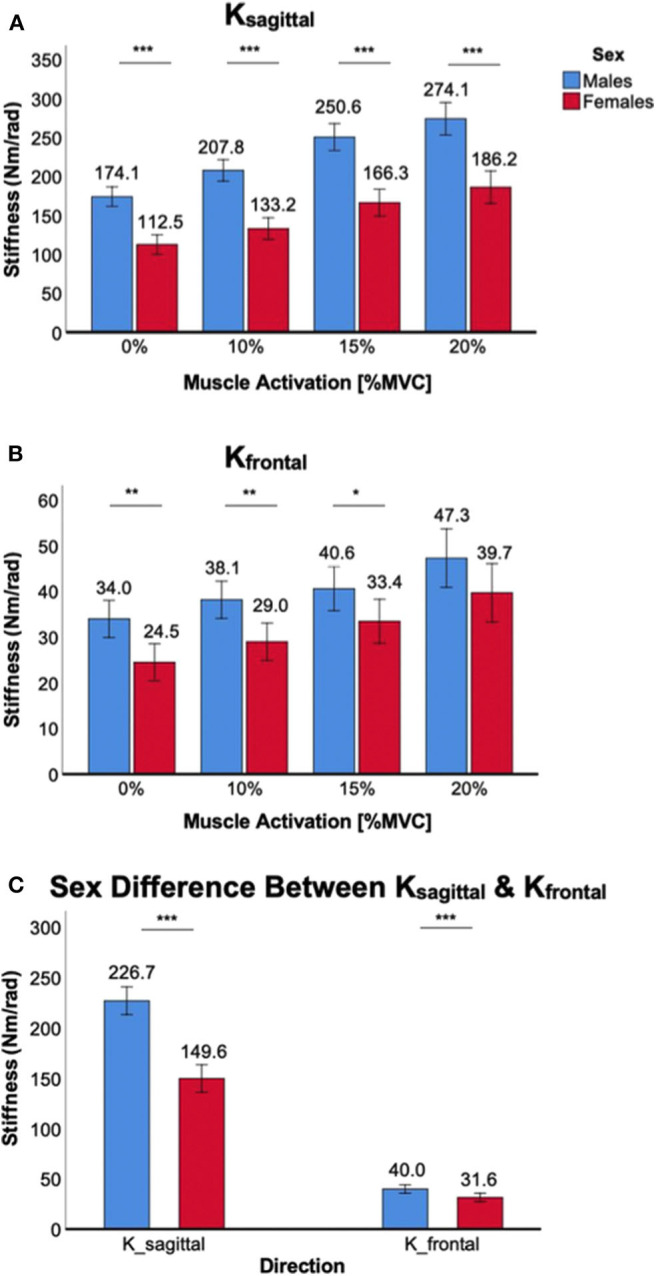
Sex differences in 2D ankle stiffness for the muscle co-contraction tasks. **(A)** *K*_*sagittal*_, **(B)** *K*_*frontal*_, **(C)** sex difference in ankle stiffness in the sagittal plane vs. frontal plane.

Co-contraction of ankle muscles significantly increased *K*_*sagittal*_ and *K*_*frontal*_ in both male and female subjects, but there was a significant sex difference in all muscle co-contraction levels, with males having higher stiffness than females (Figure 3).

For *K*_*sagittal*_, a significant main effect of the between-subjects factor of sex was identified [*F*_(1,38)_ = 64.3, *p* < 0.001]. *Post-hoc* tests revealed that sex differences were statistically significant across all muscle co-contraction levels (*p* < 0.001) with females having lower stiffness than males (Figure 3A). While a trend was observed that the sex difference in *K*_*sagittal*_ increased with increasing ankle muscle co-contraction, interaction between ankle muscle activation and sex did not reach the statistical significance [*F*_(1.6,59.9)_ = 2.6, *p* = 0.10].

For *K*_*frontal*_, a significant main effect of sex was also identified [*F*_(1,38)_ = 8.0, *p* < 0.05]. *Post-hoc* tests revealed that sex differences were statistically significant for 0% (*p* < 0.01), 10% (*p* < 0.01), and 15% MVCC (*p* < 0.05) except 20% MVCC (*p* = 0.10), with females having lower stiffness than males (Figure 3B). There was no significant interaction between ankle muscle activation and sex [*F*_(2.0,73.9)_ = 0.31, *p* = 0.73].

While the sex difference in ankle stiffness was observed in both planes of ankle motion in all muscle co-contraction levels, the difference was significantly greater in the sagittal plane than in the frontal plane (Figure 3C), which was evidenced by significant interaction between the within-subjects factor of plane of ankle motion and the between-subjects factor of sex [*F*_(1,158)_ = 93.1, *p* < 0.001].

### Sex Differences in Ankle Stiffness During Weight Bearing

**Figure 4:**
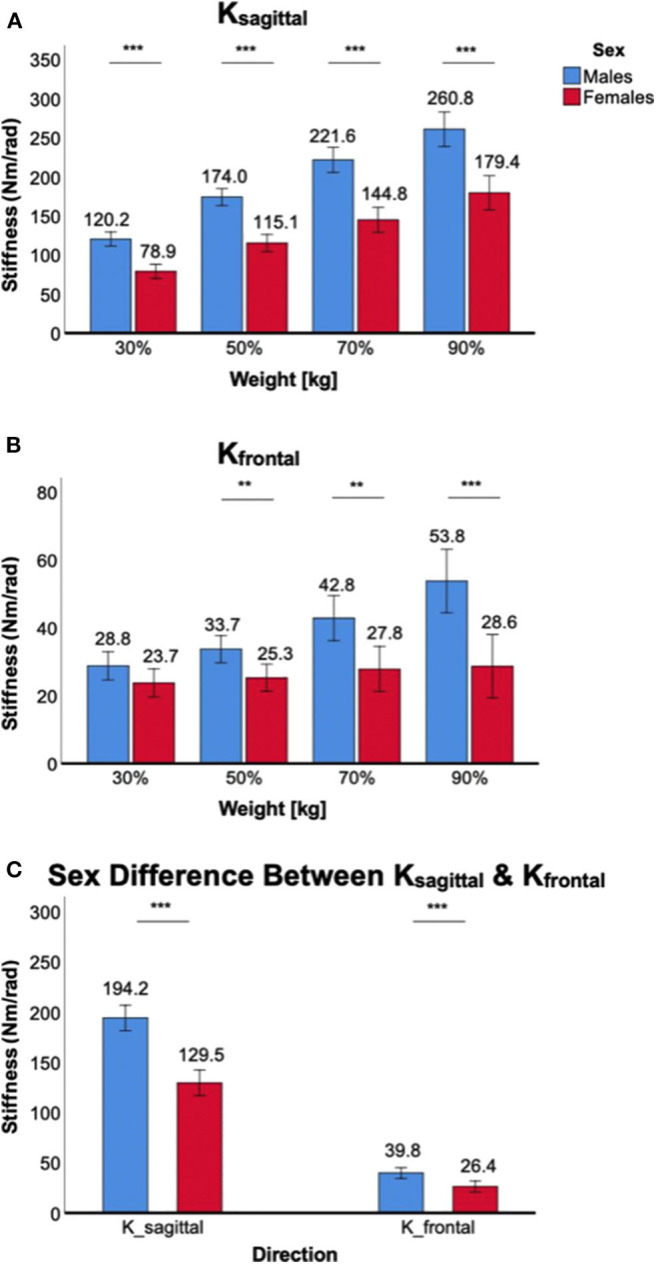
Sex differences in 2D ankle stiffness for the weight-bearing tasks. **(A)** *K*_*sagittal*_, **(B)** *K*_*frontal*_, **(C)** sex difference in ankle stiffness in the sagittal plane vs. frontal plane.

Weight-bearing at the ankle significantly increased *K*_*sagittal*_ and *K*_*frontal*_ in both male and female subjects, but there was a significant sex difference in all weight levels, with males having higher stiffness than females (Figure 4).

For *K*_*sagittal*_, a significant main effect of the between-subjects factor of sex was identified [*F*_(1,38)_ = 52.8, *p* < 0.001). *Post-hoc* tests further revealed that sex differences were statistically significant across all weight levels in the sagittal plane (*p* < 0.001) with females having lower stiffness than males (Figure 4A). There was also a significant interaction between weight-bearing and sex [*F*_(1.6,61.4)_ = 6.8, *p* < 0.05].

For *K*_*frontal*_, a significant main effect of sex was identified [*F*_(1,38)_ = 12.6, *p* < 0.01]. *Post-hoc* tests further revealed that sex differences were statistically significant across all weight levels (*p* < 0.01) except 30% body weight (*p* = 0.10), with females having lower stiffness than males (Figure 4B). There was a statistically significant interaction between weight-bearing and sex [*F*_(1.7,65.2)_ = 9.8, *p* < 0.001]. This was mainly due to little stiffness changes in females across different weight-bearing conditions.

While the sex difference in ankle stiffness was observed in both planes of ankle motion in all weight levels, the difference was significantly greater in the sagittal plane than in the frontal plane (Figure 4C). This was evidenced by significant interaction between the within-subjects factor of plane of ankle motion and the between-subjects factor of sex [*F*_(1,158)_ = 40.1, *p* < 0.001].

### Sex Differences in Ankle Stiffness During Ankle Torque Generation (via CoP Displacement)

Significant sex differences in *K*_*sagittal*_ and *K*_*frontal*_ were observed in all ankle torque generation (CoP displacement) conditions, with stiffness in males being greater than females.

**Figure 5:**
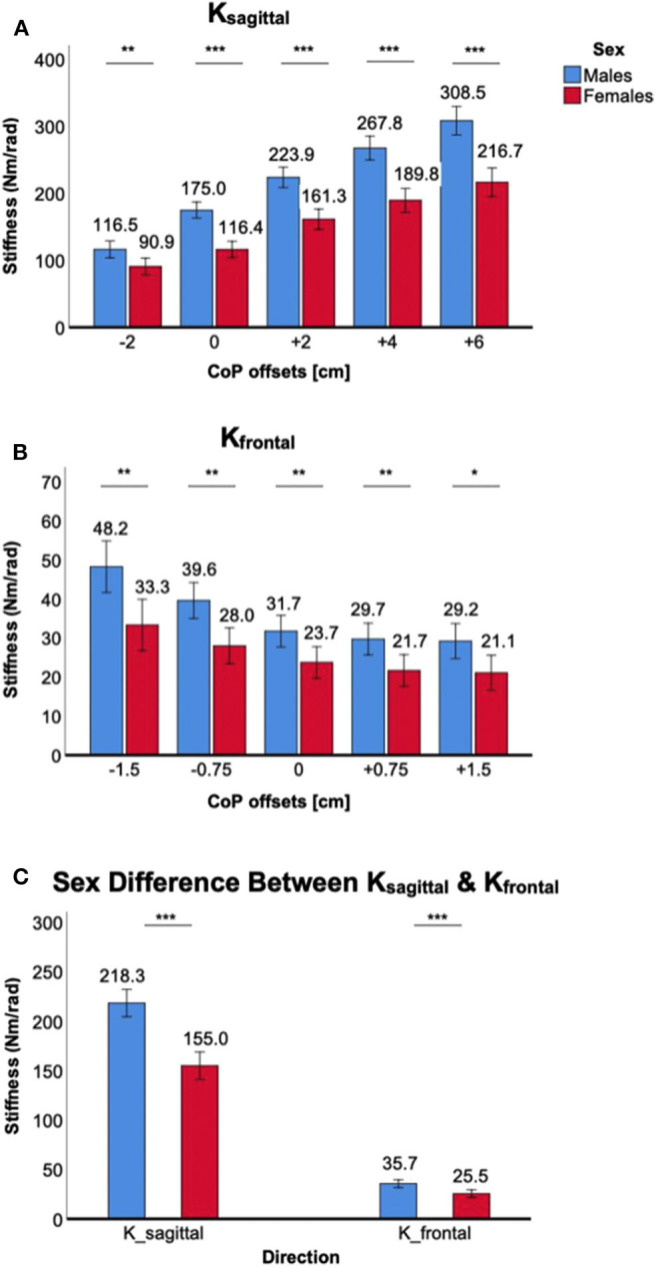
Sex differences in 2D ankle stiffness for the ankle torque generation (CoP) tasks. **(A)** *K*_*sagittal*_, **(B)** *K*_*frontal*_, **(C)** sex difference in ankle stiffness in the sagittal plane vs. frontal plane.

For *K*_*sagittal*_, a significant main effect of the between-subjects factor of sex was identified [*F*_(1,38)_ = 43.1, *p* < 0.001]. *Post-hoc* tests further revealed that sex differences were statistically significant across all torque conditions: *p* < 0.01 for −2 cm CoP and *p* < 0.001 for all other conditions, with females having lower stiffness than males (Figure 5A). There was a significant interaction between ankle torque generation in the sagittal plane and sex [*F*_(2.2,81.8)_ = 14.7, *p* < 0.05].

For *K*_*frontal*_, a significant main effect of the between-subjects factor of sex was identified [*F*_(1,38)_ = 13.9, *p* < 0.001]. *Post-hoc* tests further revealed that sex differences were statistically significant across all torque conditions: *p* < 0.05 for +1.5 cm CoP and *p* < 0.01 for all other conditions, with females having lower stiffness than males (Figure 5B). The sex difference became greater with eversion torque generation (negative CoP). However, interaction between ankle torque generation in the frontal plane and sex did not reach the statistical significance [*F*_(2.4,91.9)_ = 1.9, *p* = 0.15].

While the sex difference in ankle stiffness was observed in both planes of ankle motion in all ankle torque generation levels, the difference was significantly greater in the sagittal plane than in the frontal plane. This was evidenced by significant interaction between the within-subjects factor of plane of ankle motion and the between-subjects factor of sex [*F*_(1,198)_ = 26.5, *p* < 0.001; Figure 5C].

### Sex Differences in Normalized Ankle Stiffness

**Figure 6:**
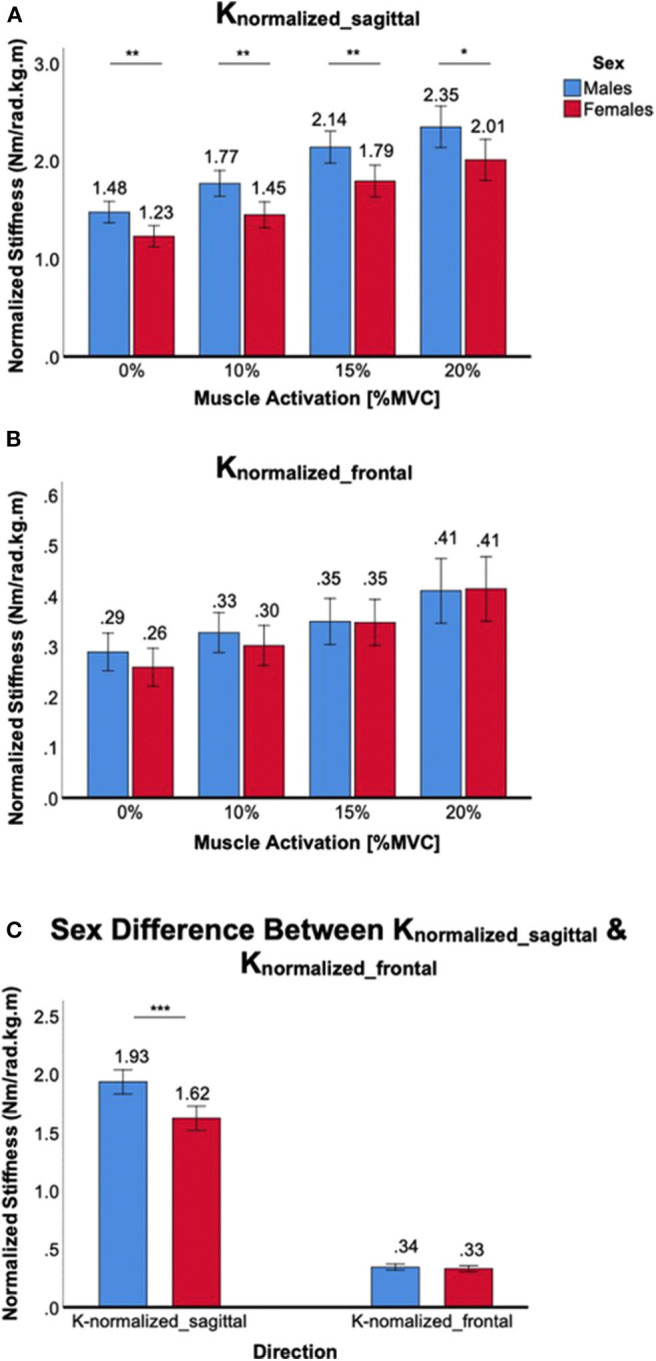
Sex differences in normalized 2D ankle stiffness for the muscle co-contraction tasks. **(A)** *K*_*normalized*_*sagittal*_, **(B)** *K*_*normalized*_*frontal*_, **(C)** sex difference in normalized ankle stiffness in the sagittal plane vs. frontal plane.

Co-contraction of ankle muscles increased normalized ankle stiffness in both male and female subjects, but there was a significant sex difference only in the sagittal plane (Figure 6). For *K*_*normalized*_*sagittal*_, a significant main effect of the between-subjects factor of sex was identified [*F*_(1,38)_ = 11.4, *p* < 0.01]. *Post-hoc t*-tests revealed that sex differences were statistically significant across all muscle co-contraction levels, with females having lower normalized stiffness than males (Figure 6A). For *K*_*normalized*_*frontal*_, there was no significant main effect of the between-subjects factor of sex [*F*_(1,38)_ = 0.2, *p* = 0.64]. In addition, *post-hoc t*-tests revealed no statistical sex differences across all muscle co-contraction levels (Figure 6B). Sex difference observed in the sagittal plane was significantly greater than in the frontal plane, evidenced by significant interaction between the within-subjects factor of plane of ankle motion and the between-subjects factor of sex [*F*_(1,158)_ = 19.5, *p* < 0.001; Figure 6C].

**Figure 7:**
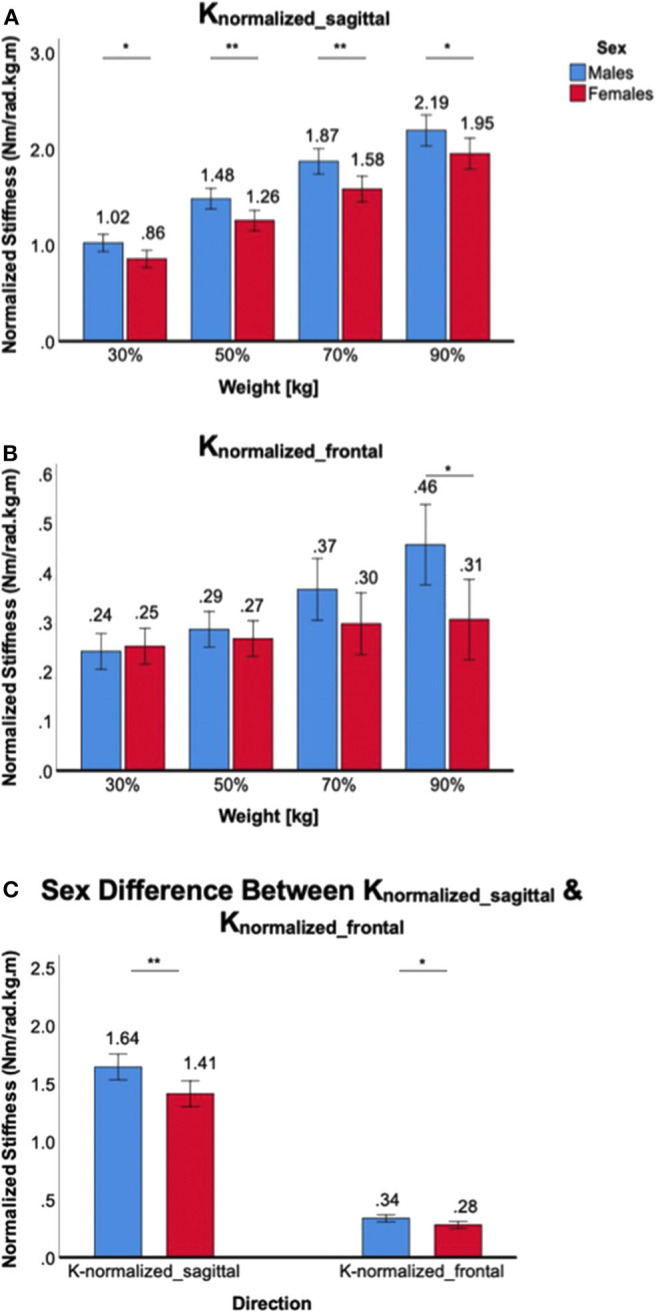
Sex differences in normalized 2D ankle stiffness for the weight-bearing tasks. **(A)** *K*_*normalized*_*sagittal*_, **(B)** *K*_*normalized*_*frontal*_, **(C)** sex difference in normalized ankle stiffness in the sagittal plane vs. frontal plane.

Increasing weight-bearing at the ankle joint increased *K*_*normalized*_*sagittal*_ in both male and female subjects, but there was a significant sex difference in all weight-bearing levels. A significant main effect of the between-subjects factor of sex was identified [*F*_(1,38)_ = 10.5, *p* < 0.01], and *post-hoc* tests revealed that sex differences were statistically significant across all weight-bearing levels, with females having lower normalized stiffness than males (Figure 7A). For *K*_*normalized*_*frontal*_, there was no significant main effect of the between-subjects factor of sex [*F*_(1,38)_ = 2.9, *p* = 0.10]. In addition, *post-hoc t*-tests revealed that no statistically significant sex differences were identified across all weight-bearing levels with the exception of 90% weight (Figure 7B). Sex difference observed in the sagittal plane was significantly greater than in the frontal plane, evidenced by significant interaction between the plane of ankle motion and sex [*F*_(1,158)_ = 5.6, *p* < 0.05; Figure 7C].

**Figure 8:**
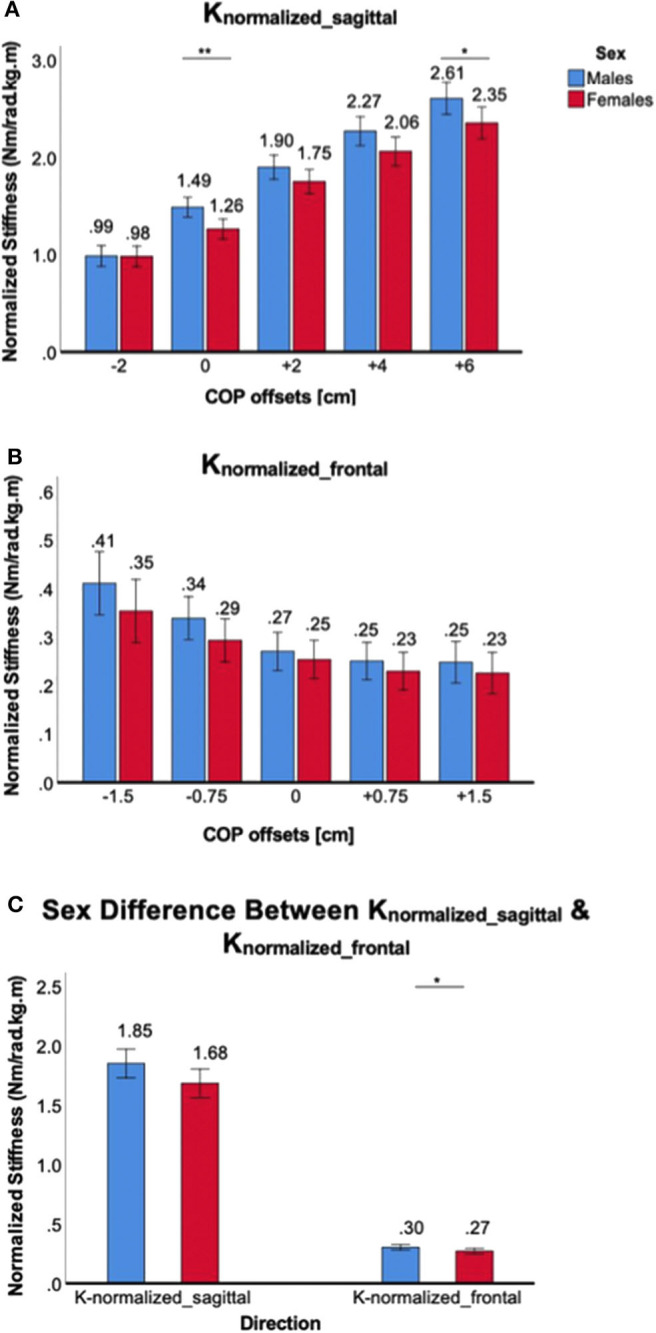
Sex differences in normalized 2D ankle stiffness for the ankle torque generation tasks. **(A)** *K*_*normalized*_*sagittal*_, **(B)** *K*_*normalized*_*frontal*_, **(C)** sex difference in normalized ankle stiffness in the sagittal plane vs. frontal plane.

For the ankle torque generation tasks, similar trends were observed in normalized ankle stiffness when compared to absolute ankle stiffness. For *K*_*normalized*_*sagittal*_, a significant main effect of the between-subjects factor of sex was identified [*F*_(1,38)_ = 5.1, *p* < 0.05]. *Post-hoc* tests revealed significant sex differences for only two conditions, namely 0 and +6 cm CoP offsets, and one marginal condition for +4 cm CoP, with females having lower normalized stiffness than males (Figure 8A). For *K*_*normalized*_*frontal*_, there was no significant main effect of the between-subjects factor of sex [*F*_(1,38)_ = 1.5, *p* = 0.22]. *Post-hoc* tests revealed no statistically significant sex difference across all weight-bearing conditions (Figure 8B). Although sex differences in the sagittal plane was greater than in the frontal plane, there was no significant interaction between the plane of ankle motion and sex [*F*_(1,198)_ = 2.05, *p* = 0.15; Figure 8C].

## Discussion

Previous studies have demonstrated that the incidence of musculoskeletal injuries at the ankle joint in females is significantly higher than in males participating in similar sports activities (Elias, 2001; Ristolainen et al., 2009). Among the risk factors contributing to this higher incidence of musculoskeletal injuries in females, the neuromuscular control of stability has been identified as one of the potential factors contributing to the sex difference in risk of injury (Granata et al., 2002a,b; Trevino and Lee, 2018). With ankle stiffness recognized as a major contributor of neuromuscular control of stability (Loram and Lakie, 2002; Lee and Hogan, 2015), one recent study investigated sex differences in 2D ankle stiffness in a seated position (Trevino and Lee, 2018). Extending this previous study (Trevino and Lee, 2018), we identified sex differences in 2D ankle stiffness in the sagittal and frontal planes during upright standing balance under various task conditions: varying ankle muscle co-contraction, weight-bearing, and ankle torque generation tasks.

While ankle stiffness increased significantly in both the sagittal and frontal planes as the level of muscle co-contraction increased, there were clear sex differences in all muscle activation conditions. During quiet standing (0% MVCC), females exhibited lower ankle stiffness in both the sagittal and frontal planes. This could be explained by the sex differences in passive resistance to joint motion and in anatomical factors, such as greater range of motion, lower Young's modulus, and the higher ligamentous laxity (Wilkerson and Mason, 2000; Beynnon et al., 2001; Kubo et al., 2003) in females than in males. The lower stiffness in females during co-contraction (10–20% MVCC) could be mostly explained by sex differences in active muscle mechanics. Previous studies have demonstrated that males have more leg muscle mass (Janssen et al., 2000), fast-twitch fibers (Glenmark et al., 2004), a higher cross-sectional area (Miller et al., 1993), and a higher rate of force production (Haizlip et al., 2015) compared to females.

Even after normalizing ankle stiffness by weight times height, *K*_*normalized*_*sagittal*_ still showed significant sex differences in all muscle co-contraction levels. It is worth to note that the overall *K*_*normalized*_*sagittal*_ in females was 19.1% lower than that in males, which is significantly lower than the difference of absolute ankle stiffness (*K*_*sagittal*_) between sexes (50.0%). Contrary to the results in the sagittal plane, *K*_*normalized*_*frontal*_ showed no statistical difference in all muscle co-contraction tasks. Based on these results, sex differences in ankle stiffness in the sagittal plane during muscle co-contraction tasks could be explained by both anthropometric factors and sex differences in neuromuscular factors. On the other hand, sex differences in ankle stiffness in the frontal plane could be mostly explained by anthropometric factors but not the neuromuscular factors.

In all weight-bearing conditions, clear sex differences were identified in both the sagittal and frontal planes. This could be primarily due to higher weight and height of males than females which translates to higher ankle torque and higher ankle stiffness in males than females. In this study, the average female weight and height was 85.6 and 96.1% of those in males. It is also worth noting that, unlike males, females showed no statistical difference in frontal plane stiffness across all weight-bearing conditions. This implies that loading the ankle in females is not as effective as co-contracting ankle muscles to increase ankle stiffness and resist external perturbations.

When normalized by weight times height, the sex differences significantly decreased compared to absolute ankle stiffness. In the frontal plane, there was no statistical sex difference in *K*_*normalized*_*frontal*_ except the highest loading condition (90%). In the sagittal plane, while there were sex differences in *K*_*normalized*_*sagittal*_ in all weight-bearing conditions, the level of statistical differences decreased compared to that in *K*_*sagittal*_. This suggests that while anthropometric factors contribute to the modulation of 2D ankle stiffness during weight bearing tasks, there is another significant factor that accounts for the sex differences in the sagittal plane, for example, active ankle mechanics.

In the ankle torque generation tasks, most subjects changed the sway angle to maintain different target CoP offsets. In the sagittal plane, increasing CoP offsets from −2 to +6 cm correlates to the increased moment arm for the applied force. This leads to the increase in ankle torque, and consequently, the increase in ankle stiffness. In the frontal plane, increasing the magnitude of CoP offsets from 0 to 1.5 cm, increases the moment arm for the applied force, thus, leading to the increase in ankle torque. With the increase in ankle torque generation, we would expect a U-shaped trend in ankle stiffness from −1.5 to +1.5 cm. However, we only observed this trend for −1.5 to 0 cm (eversion torque required), but not for 0 to +1.5 cm (inversion torque required). This trend was consistent in both males and females, implying that generating inversion torque during standing balance is not an effective strategy to increase ankle stiffness in the frontal plane. Clear sex differences were identified in all ankle torque generation conditions in both the sagittal and frontal planes. This is primarily due to different target levels of ankle torque between males and females. Even for the same value of CoP offset, heavier males require more ankle torque than females.

When normalized by weight times height, the sex differences in ankle stiffness were reduced significantly. Although the ANOVA analysis confirmed a significant main effect of sex and there was a consistent trend of greater *K*_*normalized*_*sagittal*_ in males than in females except the −2 cm CoP condition, pairwise comparison for each CoP condition showed that only two conditions in *K*_*normalized*_*sagittal*_ reached the statistical significance. None of the conditions in *K*_*normalized*_*frontal*_ was statistically different. This is somewhat expected as ankle torque is proportional to the normalization factor of weight times height in the inverted pendulum model that incorporate postural sway for standing balance (Morasso and Schieppati, 1999; Winter et al., 2001). Thus, sex differences in ankle stiffness in the sagittal plane during ankle torque generation via sway angle changes could be largely explained by anthropometric factors while there still exist non-negligible contribution of other neuromuscular factors. The differences in the frontal plane could be mostly explained by anthropometric factors but not the neuromuscular factors.

For all the three different tasks, ankle stiffness in the sagittal plane was significantly higher than in the frontal plane, which indicates that the ankle is relatively vulnerable to perturbations in the frontal plane. This is not surprising because ankle movement predominantly occurs in the sagittal plane, with most of the ankle muscles contributing to movement in the sagittal plane whereas a few contribute to movement in the frontal plane (Brockett and Chapman, 2016). Consequently, the range of ankle torque generation and ankle stiffness modulation is significantly higher in the sagittal plane than in the frontal plane (Lee et al., 2014a,c; Lee and Hogan, 2015), and sex differences in ankle stiffness are more amplified in the sagittal plane than the frontal plane. Even when ankle stiffness was normalized by weight times height, the sex differences in the sagittal plane was greater than in the frontal plane, implying that not only the anthropometric factor but also neuromuscular factors contribute to the direction-dependent sex differences in ankle stiffness.

The incidence of musculoskeletal injuries at the ankle joint is an ever-increasing problem especially for the female population, yet there is little information on neuromuscular basis for this higher risk of injury. This study, for the first time, investigated the sex differences in 2D ankle stiffness, one major contributor to neuromuscular control, during various standing balance tasks. Results in this study confirmed that females have significantly lower ankle stiffness during upright standing balance thereby providing the neuromuscular basis for further investigations on sex differences in 2D ankle stiffness during dynamic tasks such as walking and running as well as on the correlation of ankle stiffness and the higher risk of ankle injury in females. Outcomes from this further investigation will shed more light on the understanding of whether sex difference in ankle stiffness prospectively influences the sex difference in lower body stability and risk of ankle injury. Furthermore, they would serve as a basis to develop sex-specific training programs for effective ankle injury prevention and rehabilitation and to improve ankle performance.

## Data Availability Statement

The raw data supporting the conclusions of this article will be made available by the authors, without undue reservation.

## Ethics Statement

The studies involving human participants were reviewed and approved by Arizona State University. The patients/participants provided their written informed consent to participate in this study. Written informed consent was obtained from the individual(s) for the publication of any potentially identifiable images or data included in this article.

## Author Contributions

Conception was by HL. Design of the experiments was by HL and VN. Experiments were directed by HL and performed by VN and EA. Data analysis and interpretation of data were done by all authors. All authors contributed to the main manuscript text and approved the final version of the manuscript.

## Conflict of Interest

The authors declare that the research was conducted in the absence of any commercial or financial relationships that could be construed as a potential conflict of interest.
